# New Drugs, Appliances, and Things Medical

**Published:** 1893-03-25

**Authors:** 


### NEW DRUGS, APPLIANCES, AND THINGS MEDICAL.

[All preparations, appliances, novelties, etc., of which a notice is desired, should be sent for the Editor, to care of The Manager, 428, Strand, London, W.C.]

### "DORINA" NURSERY BISCUITS.

(Messrs. Chibnall, King Street, Hammersmith.)

Messrs. Chibnall, of Hammersmith, have made a specialty of biscuits suitable for infants, invalids, and dyspeptics. In appearance and taste they resemble the rusks known as "tops and bottoms," which are intimately connected with the remembrance of our nursery days. In form they are more dainty, of a more serviceable size, and are so palatable that their use is likely to spread beyond the region of the nursery and sick room. They have nevertheless been prepared with great care to form digestive and nutritious food for infants and invalids. The aim of the manufacturer has been carried out, and these rusks or biscuits contain nearly 20 per cent. more soluble matter than ordinary farinaceous commodities. They contain sufficient carbo-hydrates, are rich in phosphates, and do not contain cane sugar. All these qualities especially recommend the "Dorina" nursery biscuits for use as a diet for infants and young children, whilst they are so pleasant to the taste as to prove an addition very welcome to invalids.

### DR. NANSEN AND BOVRIL.

Dr. Nansen intends to "do" his North Pole expedition on Bovril. On the invitation of Lord Playfair and the directors of Bovril we had the opportunity of inspecting samples of the different varieties of Bovril food prepared by the company for the new Arctic expedition, and also of examining their preparations generally in the course of their manufacture. A visit to the Bovril factory at Bath Street, in the City Road, impresses the critic very favourably. The place is particularly, spotlessly clean, and everybody and everything in it is clean. There are about 200 workpeople employed, and the young women and girls were as neat and orderly as a battalion of hospital nurses. The foods prepared for Dr. Nansen were all of the highly "compressed" variety. Of these there were several kinds, but the staple in each was Bovril. There were "Bovril with potatoes, vegetables, and fats," "Bovril with barley, potatoes, and ham fat," "Bovril with anchovies and oatmeal," and so on. These foods are, as far as possible, quite free from water. They are so concentrated that a few ounces a day will, it is said, support life and health in the severest of Arctic weather. We had the curiosity to taste most of them, and it is surprising how palatable they were, even though highly charged with fat and concentrated to such a marked degree. It is easy to imagine that, when mixed with boiling water and eaten with bread, they will form most appetising soups. Even in the solid form they will doubtless be relished by appetites sharpened with cold and hungry for the heat-producing fat. There were also exhibited compressed teas, Bovril wine, Bovril chocolate—a most agreeable confection—and dried fruits, vegetables, eels, and eggs. The exhibition was of the most physiologically satisfactory kind, and almost made one wish to join Dr. Nansen's expedition for the sake of enjoying his fare.

